# Polyethylene nanoplastics intensify toxicity of potassium clavulanate in African catfish (*Clarias gariepinus*)

**DOI:** 10.1038/s41598-025-27780-6

**Published:** 2025-12-03

**Authors:** Walied A Kamel, Alaa El-Din H. Sayed, Shaimaa Kamal A. Idriss, Heba Allah M. Elbaghdady

**Affiliations:** 1 Laboratory of Fundamental Oncology, National Cancer Center Research Institute, Tsukiji 5-1-1, Chuo-ku, Tokyo, 104-0045 Japan; 2 Department of Zoology, Faculty of Science, Mansoura University, Mansoura, 35516 Egypt; 3 Department of Zoology, Faculty of Science, Assiut University, Assiut, 71516 Egypt; 4 Molecular Biology Research and Studies Institute, Assiut University, Assiut, 71516 Egypt; 5 Department of Aquatic Animal Medicine and Management, Faculty of Veterinary Medicine, Assiut University, Assiut, 71516 Egypt

**Keywords:** Polyethylene nanoplastics, Potassium clavulanate, Toxicity, *Clarias gariepinus*, Zoology, Environmental sciences

## Abstract

Nanoplastics (NPs) and antibiotics frequently co-occur in aquatic environments, yet their combined effects on fish health remain poorly understood. This study examined the individual and joint toxicity of polyethylene nanoplastics (PE-NPs; 20 and 80 nm, 5 mg L⁻¹) and potassium clavulanate (CA; 100 ng L⁻¹) in African catfish (*Clarias gariepinus*) during a 15-day exposure followed by a 15-day recovery phase. Exposure to PE-NPs (20 nm) alone significantly increased erythrocyte apoptosis (2.8-fold vs. control, *p* < 0.001), while CA alone also caused significant DNA damage (1.9-fold vs. control, *p* < 0.001). PE-NPs (80 nm) alone induced no notable alterations. Co-exposure to CA and PE-NPs, particularly the 20 nm particles, produced the strongest erythrocyte apoptosis, DNA damage, and declines in Hb, Hct, RBCs, and WBCs. These hematological and genotoxic effects persisted after recovery, indicating incomplete or delayed reversal of toxicity. Spleen histopathology showed vascular congestion, hemorrhage, fibrosis, and lymphoid depletion, especially in co-exposed fish. The results demonstrate a statistically significant, size-dependent synergistic interaction, suggesting that smaller PE-NPs amplify CA-induced cytotoxicity and genotoxicity through a “Trojan horse” mechanism. The persistence of these effects underscores the ecological risk of NP–pharmaceutical mixtures and the need to include such interactions in aquatic risk assessments.

## Introduction

 Plastic pollution has become a major global concern due to its persistence, ubiquity, and adverse biological effects in aquatic ecosystems^1^. Plastics enter water bodies through wastewater discharge, littering, and coastal activities, with polyethylene, polypropylene, and polystyrene being the most common polymers^2,3^. Global plastic accumulation is projected to reach 250 million tons by 2025, underscoring the need to assess its ecological and toxicological impacts^4^.

Based on particle size, plastics are classified as macro-, meso-, micro- (5 mm–100 nm), and nanoplastics (< 100 nm), which can be ingested and transferred through food webs^5^. Nanoplastics (NPs) are particularly concerning because of their nanoscale size and large surface area, which enable cellular penetration and the induction of oxidative stress^6–8^. Among them, polyethylene nanoplastics (PE-NPs) are widespread and persistent, capable of triggering oxidative imbalance, lipid peroxidation, and immune dysfunction in fish^9–11^. Smaller PE-NPs generally exhibit higher toxicity due to increased surface reactivity and cellular uptake^12^.

In aquatic environments, NPs frequently interact with other contaminants such as heavy metals, pesticides, and pharmaceuticals, altering their transport, bioavailability, and toxicity through surface adsorption or “Trojan horse” effects^13–15^. Pharmaceuticals, particularly antibiotics and β-lactamase inhibitors, are of growing concern due to their continuous input from domestic and hospital wastewater and their persistence in surface waters^16–23^. Among them, potassium clavulanate (CA), commonly co-administered with β-lactam antibiotics, is increasingly detected in aquatic systems at ng L⁻¹–µg L⁻¹ concentrations^24–27^. Although CA itself has limited antibacterial activity, it can cause oxidative stress, hematological alterations, and genotoxicity in fish under prolonged exposure^26,27^.

The African catfish (*Clarias gariepinus*) is a widely used bioindicator species because of its ecological importance, environmental tolerance, and physiological sensitivity to pollutants^28,29^. Its benthic feeding behavior and close association with sediments make it especially vulnerable to micro- and NPs exposure^30,31^. Therefore, *C. gariepinus* provides an appropriate model for evaluating the combined effects of PE-NPs and pharmaceuticals in freshwater ecosystems.

While the individual toxicities of PE-NPs and CA have been documented, their combined effects remain poorly understood in ecologically and economically important fish such as *C. gariepinus*^32–34^. Previous NP–antibiotic studies have examined polystyrene NPs combined with antimicrobials such as sulfamethoxazole or enrofloxacin in small laboratory models (e.g., zebrafish, *Daphnia*), typically focusing on single endpoints and lacking recovery-phase assessments^32,35^.

To address these gaps, this study is novel in four key ways: (A) it examines PE-NPs, a polymer more environmentally relevant than polystyrene; (B) it compares 20 nm and 80 nm particles to test size-dependent uptake and toxicity; (C) it evaluates interactions with CA, an under-studied antibiotic, highlighting potential synergistic effects likely via a Trojan horse mechanism; and (D) it employs a comprehensive suite of endpoints—including apoptosis, DNA damage, hematology, and spleen histopathology—together with a 15-day recovery phase, providing insights into the persistence of combined contaminant effects. Unlike previous studies in small model species focusing on single endpoints, this approach offers a more ecologically and environmentally relevant assessment of NP–antibiotic co-toxicity in *C. gariepinus*.

## Materials and methods

### PE-NPs and potassium clavulanate

The PE-NPs were obtained from Micro Powders Inc. (580 White Plains Rd, Tarrytown, NY 10591, USA). The specific product, MP P-635XF (CAS No. 9002-88-4), consisted of white powder with an average particle size ranging from 20 to 100 nm. The stock solution was prepared by dispersing PE-NPs in deionized water using a magnetic stirrer at room temperature. Before modifying or combining the NPs with any other substance, their structure was examined and recorded using a scanning electron microscope at the transmission electron microscope (TEM) unit of Assiut University (JEOL JEM-1200 EX II, Massachusetts, USA). The NPs’ composition was confirmed using Fourier transform infrared (FTIR) spectroscopy (Thermo Scientific Nicolet iS10) in the range of 4000–400 cm⁻¹. The sizes of the PE-NPs selected for this study (20 nm and 80 nm) were verified by TEM in a prior investigation^36^.

Although NPs are generally detected in aquatic environments at ng–µg/L levels, localized hotspots such as wastewater effluents, aquaculture systems, and river mouths receiving high plastic loads may reach considerably higher concentrations. To model a high-exposure scenario and ensure measurable biological responses within the 15-day experimental window, 5 mg L⁻¹ PE-NPs (20 and 80 nm) were selected as the test concentrations. This approach is consistent with previous ecotoxicological studies that used mg L⁻¹ NPs exposures to investigate mechanistic toxicity and interactions with co-contaminants in fish and invertebrate models^37–42^.

Similarly, CA has demonstrated cytotoxic effects in *Perna perna* mussels at 50–100 ng L⁻¹, closely matching the level applied in this investigation^43^. The chemical stability of both compounds was also considered: the PE-NPs suspension was maintained in a stable and homogeneous state throughout exposure, and for CA, its known hydrolytic instability in aqueous solutions^43^ was addressed by ensuring exposures were conducted under conditions minimizing degradation. These methodological considerations confirm that the applied concentrations of both PE-NPs and CA were reliable and representative of the intended treatments.

### Fish

Early juvenile male *C. gariepinus* were used, with lengths ranging from 10 to 15 cm and weights from 100 to 150 g. The fish were fed a commercial pellet diet at 3% of their body weight per day and acclimated for two weeks in water with controlled conditions: temperature (28.5 °C), conductivity (260.8 µS/cm), dissolved oxygen (6.9 mg L⁻¹), light: dark cycle (12:12 h), and pH (7.4). The water was renewed daily (50% volume exchange).

Fish were exposed to CA, PE-NPs (20–80 nm), and their combinations for 15 days, followed by a 15-day recovery phase, consistent with prior fish toxicology protocols^44^. This duration was chosen because comparable recovery periods (14–15 days) have shown that hematological, biochemical, and histopathological responses either stabilize or persist within this timeframe^11,45^, providing a reliable window for assessing reversibility or persistence of toxic effects.

At the end of each exposure and recovery period, six fish were randomly collected from each tank for genotoxic, hematological, and histopathological analyses. Fish were gently netted to minimize handling stress, anesthetized on ice, and sampled in a balanced manner across replicate tanks^46^. Blood was collected from the caudal vein for hematological analysis. The Research Ethics Committee of the Faculty of Science, Assiut University, Assiut, Egypt, approved the experimental design and fish handling protocols.

### Experimental design

#### Sample distribution

- After the acclimation period, fish were randomly distributed into six groups.
- Each group contained 30 fish (10 per replicate, three replicates per group).
- All groups were maintained in glass tanks containing 100 L of water under identical conditions.

#### Treatment groups

- Group I: Control (no exposure).
- Group II: CA (100 ng L⁻¹) for 15 days.
- Group III: PE-NPs (20 nm, 5 mg L⁻¹) for 15 days.
- Group IV: PE-NPs (80 nm, 5 mg L⁻¹) for 15 days.
- Group V: CA + PE-NPs (20 nm, 5 mg L⁻¹) for 15 days.
- Group VI: CA + PE-NPs (80 nm, 5 mg L⁻¹) for 15 days.

This design enabled comparison of the effects of CA, PE-NPs of different sizes, and their combinations, with the control group serving as the baseline.

### Genotoxic biomarkers

Acridine orange staining (Cat. No. A1031, Life Technologies, Carlsbad, CA, USA) was used to detect apoptotic erythrocytes as described by^47^. Blood smears were prepared on clean glass slides, rinsed with 1× phosphate-buffered saline (PBS, pH 7.2), and stained with an AO buffer containing 17 µg/L acridine orange for 30 min in darkness. The slides were decolorized by washing with PBS every 30 min (four times), fixed in 4% paraformaldehyde for 5 min, and examined under a Zeiss Axioplan2 fluorescence microscope at 200× magnification.

DNA damage was assessed using the comet assay according to Sayed et al.^48^. Blood (3 µL) was collected from the caudal vein and placed on ice to prevent repair processes. Only samples with > 90% cell viability were analyzed. Sixty cells per slide were examined under a fluorescence microscope (Zeiss Axioplan2) equipped with a digital camera (Sony, AVT Horn, Japan). Tail moment scores were calculated using Comet Assay Software Project.

### Hematological parameters

Following the 15-day exposure, six fish per group were anesthetized on ice, and blood was collected from the caudal vein using a 1 mL syringe. Part of each sample was placed in tubes containing anticoagulant for hematological analysis. Parameters measured included red blood cells (RBCs), white blood cells (WBCs), hemoglobin (Hb) concentration, hematocrit (HCT), mean corpuscular volume (MCV), mean corpuscular hemoglobin (MCH), mean corpuscular hemoglobin concentration (MCHC), platelets, neutrophils, lymphocytes, monocytes, and eosinophils, determined using an automatic hematology analyzer.

### Statistical analysis

Data were analyzed using GraphPad Prism 5 (GraphPad Software, CA, USA). One-way ANOVA followed by Tukey’s post hoc test was used. Data are presented as mean ± SEM. Results with *p* > 0.05 were considered non-significant; those with *p* < 0.05, *p* < 0.01, and *p* < 0.001 were considered significant, highly significant, and very highly significant, respectively.

### Tissue sections for histopathological studies

Following a 3-day and 15-day experimental interval, fish were gathered from each tank. The spleen was directly dissected from each fish and subsequently rinsed with a neutral saline solution. Each tissue specimen was preserved in neutral buffered formaldehyde, then dehydrated using a series of ethanol alcohol solutions, followed by clearing with methyl benzoate, and finally embedded in paraffin wax^49^. Prepare thin pieces with a thickness of 5 microns. Following dewaxing and rehydration, the slides were stained with Hematoxylin and Eosin (H & E Masson Trichrome’s and Sirius Red stains, following the established methodology^50^. The Olympus CH30 microscope was used to analyze and photograph all sections histopathologically^51^. The severity of the pathology was assessed using the following scale: (-) for absence, (+) for minimal, (++) for moderate, (+++) for severe, and a maximum score of (++++)^51^.

## Results

### The principle of the study

**Fig. 1:**
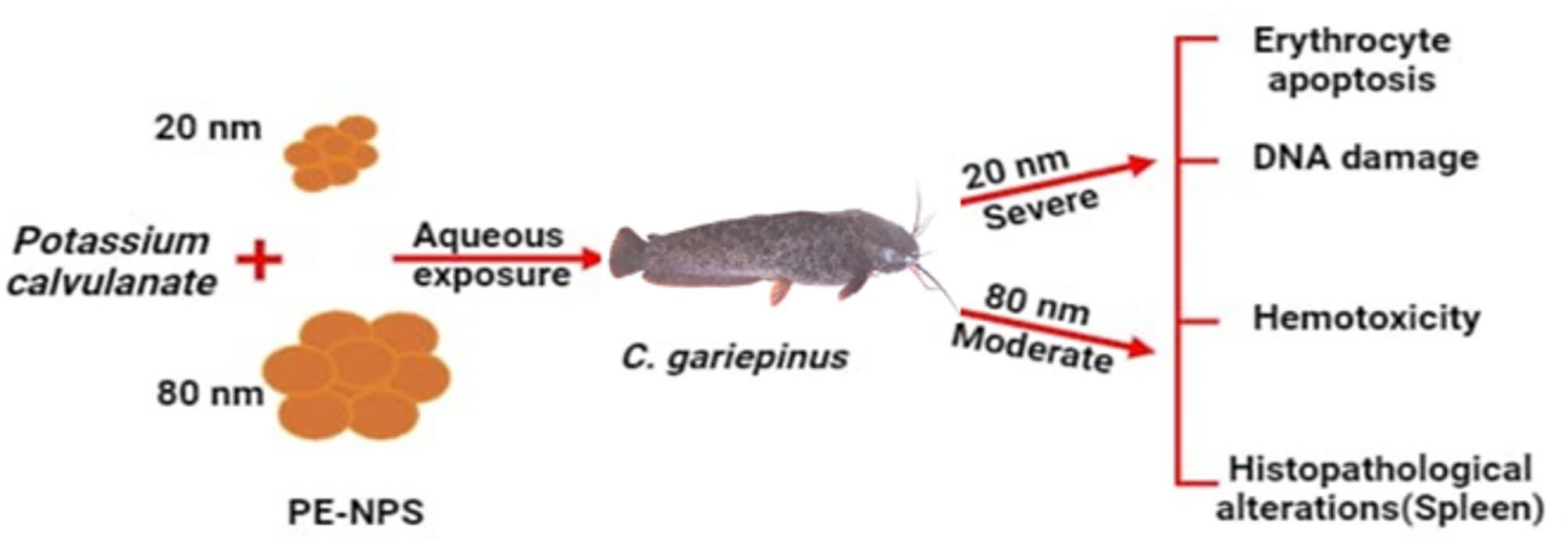
Experimental design of the study. *C. gariepinus* were exposed for 15 days to CA, 100 ng L⁻¹, PE-NPs, 5 mg L⁻¹ (20–80 nm), or their combinations, followed by a 15-day recovery period. Endpoints evaluated included erythrocyte apoptosis, DNA damage, hematological parameters, and spleen histopathology.

The experimental design is illustrated in Fig. 1. The toxic effects were evaluated by exposing *C. gariepinus* to 100 ng L⁻¹ CA, 5 mg L⁻¹ PE-NPs (20 nm), and 5 mg L⁻¹ PE-NPs (80 nm), both individually and in combination, for 15 days, followed by a 15-day recovery period. Apoptosis and DNA damage were assessed to evaluate cellular responses, while hematological and splenic alterations were analyzed to determine systemic and tissue-level effects.

### Physicochemical characterization of PE-NPs

**Fig. 2:**
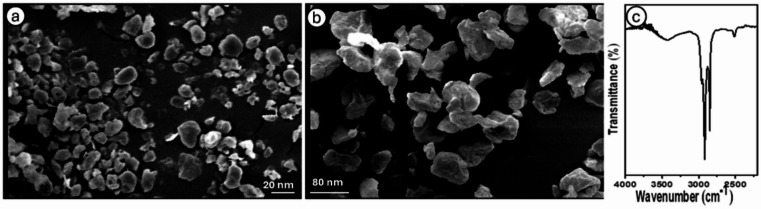
Physicochemical characterization of PE-NPs. (**a**) Scanning electron microscope (SEM) image of PE-NPs (20 nm). (**b**) SEM image of PE-NPs (80 nm). (**c**) An investigation of PE-NPs using FTIR was conducted throughout the wavenumber range of 4000–400 cm^− 1^.

The PE-NPs examined in this study consisted of polydisperse, irregular spherical aggregates with distinct sharp edges (Fig. 2a, b). The chemical structure of PE-NPs was confirmed by FTIR analysis within the wavelength range of 4000–400 cm⁻¹. As a result of the methylene (CH₂) symmetric (δs) and antisymmetric (δas) stretching vibrations, characteristic peaks were observed at 2848.86 and 2922.16 cm⁻¹, respectively (Fig. 2c)^52^.

Due to their unique physicochemical properties—particularly size and surface characteristics—it was essential to evaluate the potential contribution of PE-NPs to cellular and molecular toxicity in *C. gariepinus*.

### Genotoxic biomarkers for DNA damage

**Fig. 3:**
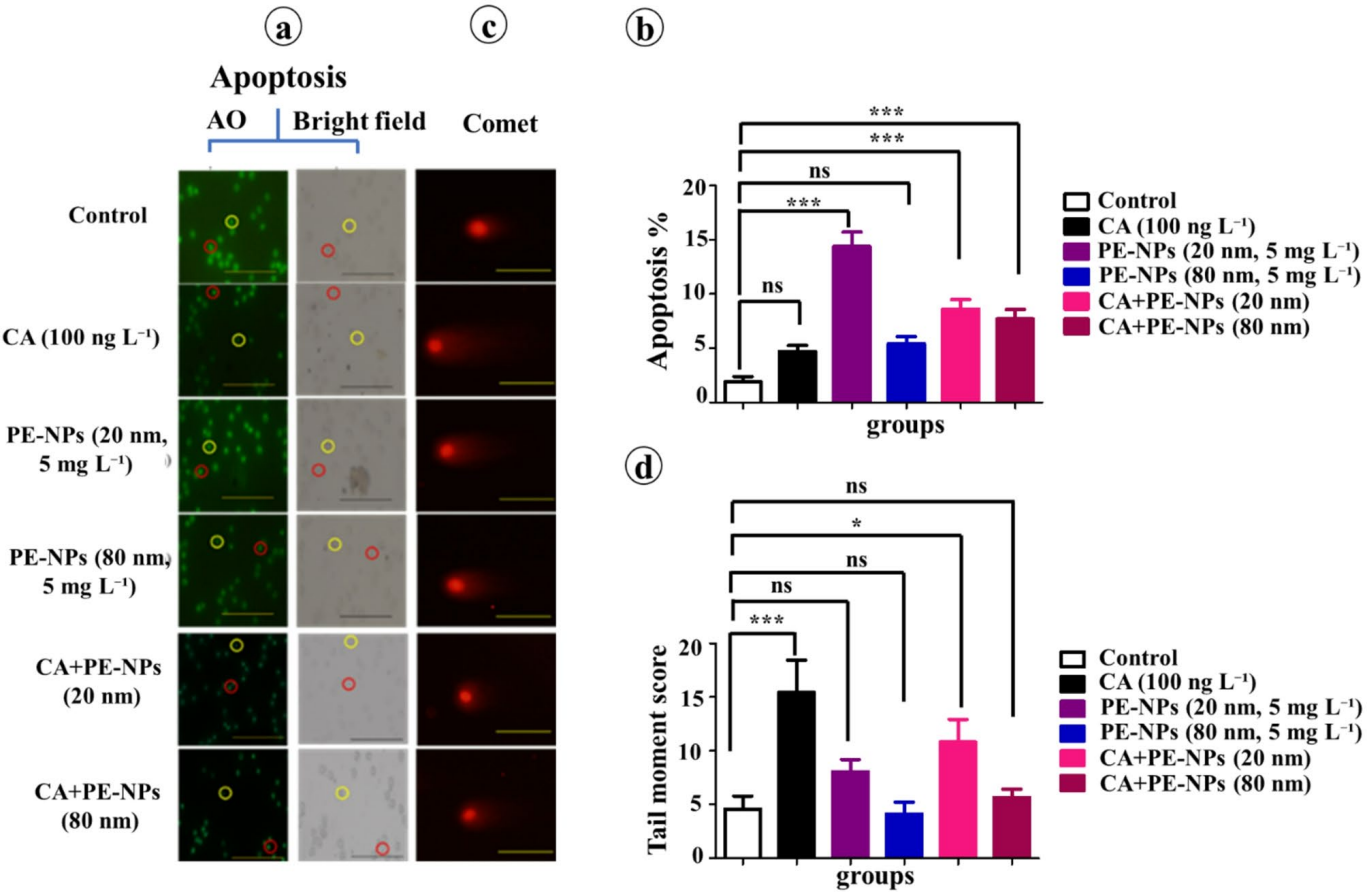
Apoptosis and DNA damage in erythrocytes of *C. gariepinus*. after 15-day exposure. Fish were exposed to CA (100 ng L⁻¹), PE-NPs (5 mg L⁻¹; 20–80 nm), or their combinations. (**a**) Representative fluorescence images of apoptotic erythrocytes (red circles) and non-apoptotic erythrocytes (yellow circles). (**b**) Percentage of apoptotic cells. (**c**–**d**) DNA damage assessed by comet assay (tail moment score) (scale bar = 50 μm). Data are shown as mean ± standard error (SE) (*n =* 6). **Statistical significance: *p < 0.05, ***p < 0.001; ns = non-significant.

Exposure to PE-NPs (20 nm) significantly increased erythrocyte apoptosis in *C. gariepinus* (2.8-fold vs. control, *p* < 0.001). In contrast, CA alone and PE-NPs (80 nm) showed no significant changes compared with the control. When combined with CA, both PE-NPs sizes significantly enhanced the apoptotic response compared with the control (*p* < 0.001), with 20 nm particles exerting stronger pro-apoptotic than 80 nm particles (Fig. 3a, b). These results demonstrate a clear size-dependent effect and support a synergistic mechanism, whereby PE-NPs intensify CA-induced cellular toxicity.

Comet assay analysis revealed that CA alone significantly increased DNA damage (1.9-fold vs. control, *p* < 0.001). PE-NPs alone caused a non-significant increase; however, the CA + PE-NPs (20 nm) group showed a significant rise in DNA fragmentation (1.4-fold vs. control, *p* < 0.05). No significant enhancement was detected with CA + PE-NPs (80 nm), indicating that the apparent difference for 80 nm particles represents a descriptive trend rather than a statistically supported effect (Fig. 3c–d).

Taken together, these data confirm that the size-dependent interaction was statistically evident only for the 20 nm particles, while 80 nm effects remained non-significant.

**Fig. 4:**
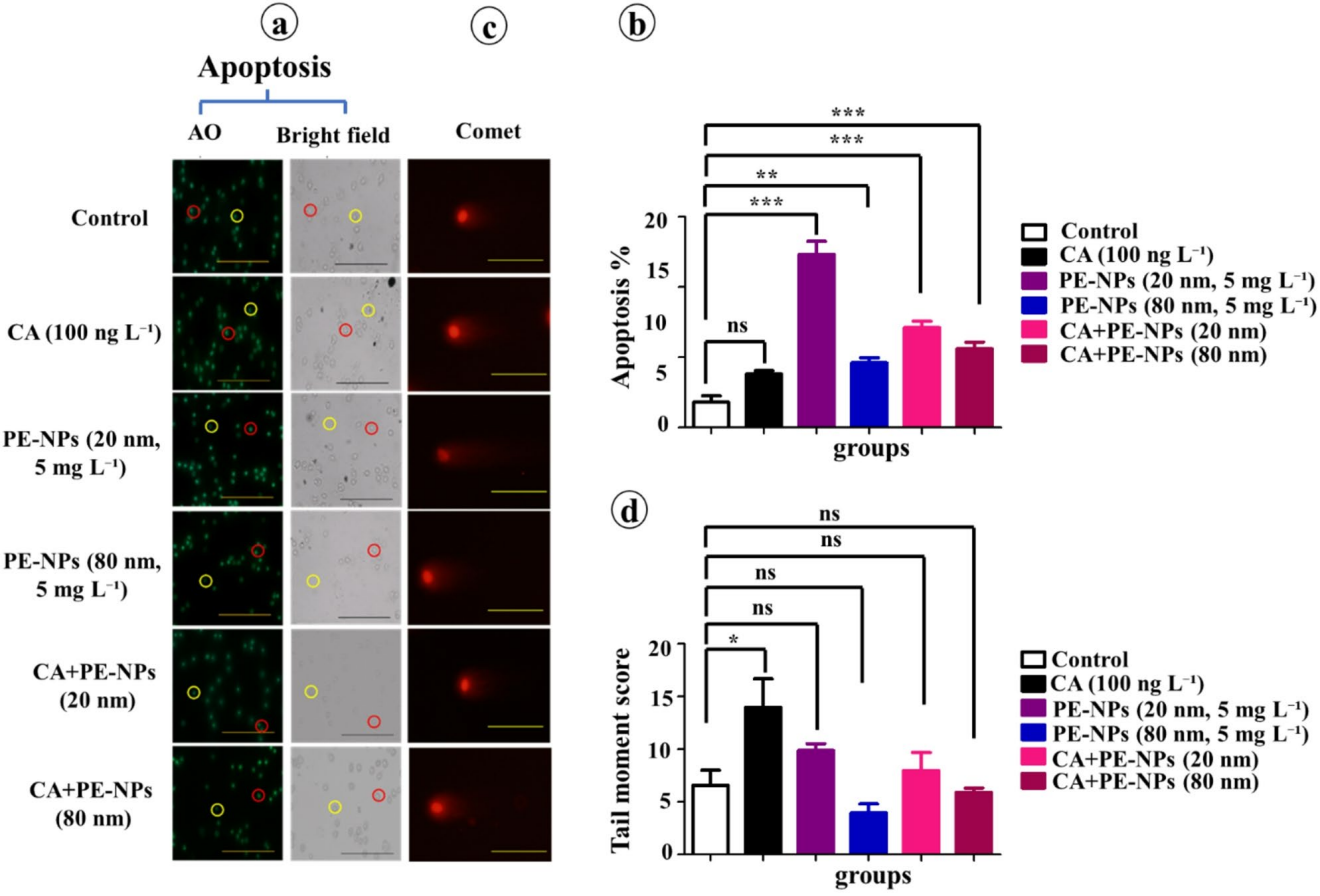
Apoptosis and DNA damage in erythrocytes of *C. gariepinus* after a 15-day recovery period. Fish previously exposed to CA (100 ng L⁻¹), PE-NPs (5 mg L⁻¹; 20–80 nm), or their combinations were evaluated following recovery. (**a**) Fluorescence images of apoptotic (red circles) and non-apoptotic (yellow circles) erythrocytes. (**b**) Percentage of apoptotic cells. (**c**–**d**) DNA damage quantified by comet assay (tail moment score) (scale bar = 50 μm). Data are presented as mean ± standard error (SE) (*n =* 6). **Statistical significance: *p < 0.05, **p < 0.01, ***p < 0.001; ns = non-significant.

Following the 15-day recovery period, apoptosis remained elevated, particularly in the CA + PE-NPs (20 nm and 80 nm) groups (Fig. 4a–d). These findings suggested that the toxic effects of PE-NPs, especially in combination with CA, may not be fully reversible.

### Effect on hematological parameters

Exposure to CA alone significantly reduced HCT (28%), neutrophils, lymphocytes, monocytes, and eosinophils compared with controls (*p* < 0.05–0.001) (Figs. 1 and 2; Tables 1 and 2). Exposure to PE-NPs (20 nm) produced marked hematotoxicity, with significant reductions in Hb, HCT, RBCs, WBCs, platelets, neutrophils, lymphocytes, and eosinophils (20–40%, *p* < 0.05–0.001) compared with controls. By comparison, PE-NPs (80 nm) caused milder but still significant decreases in several parameters, particularly Hb, HCT, and lymphocytes (15–30% reduction, *p* < 0.05–0.001). Combined exposures amplified these effects. Both CA + PE-NPs groups showed pronounced decreases in Hb, HCT, WBCs, lymphocytes, and eosinophils (35–45%, *p* < 0.001). The CA + PE-NPs (20 nm) group exhibited the most severe declines, with RBCs and neutrophils reduced by 45–50% (*p* < 0.001), exceeding the effects observed with CA + PE-NPs (80 nm).

After the 15-day recovery phase, hematological parameters remained impaired in all treated groups, especially in the CA + PE-NPs (20 nm) group, which continued to show reductions across nearly all blood cell types. Some parameters (e.g., monocytes, platelets) showed only partial recovery, while complete restoration was not observed.

Overall, smaller PE-NPs (20 nm) consistently caused stronger and more persistent hematotoxicity than 80 nm particles, particularly when combined with CA.

### Histopathological changes

**Fig. 5:**
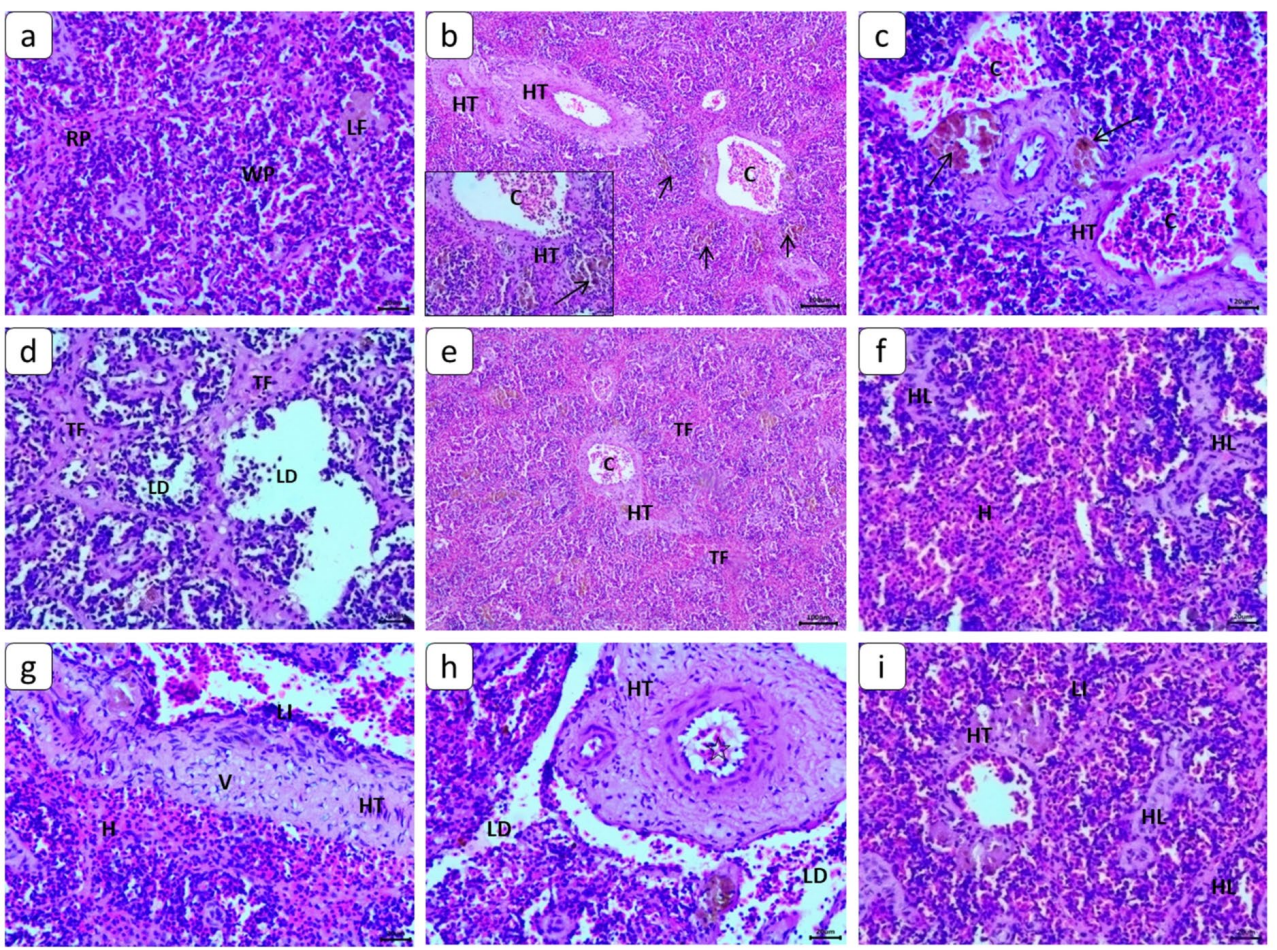
Photomicrographs of *C. gariepinus* spleen sections (H&E staining). Normal histological structure of the control group, red pulp (RP), white pulp (WP), and lymphoid follicle (LF) (**a**). In CA (100 ng L⁻¹) group, hypertrophy of tunica media (HT), MMC aggregation (arrow), and severe dilatation and congestion of blood vessels (C) were noticed (**b** and **c**). After exposure to (20 nm, 5 mg L⁻¹) & (80 nm, 5 mg L⁻¹) of PE-NPs groups moderate fibrosis of trabeculae (TF) and blood vessel wall (HT) with congestion (C) and severe depletion of lymphocytes (LD) (**d** and **e**). After exposure to CA and both sizes of PE-NPs groups, hypertrophy of tunica media (HT) with massive vacuolation (V), congestion (C), hemorrhages in red pulp (H), hyperplasia of the lymphoid follicle (HL), and massive perivascular infiltration of lymphocytes (LI) (**f** and **g**). The mild thrombosis (star) with massive hypertrophy of tunica media (HT) and severe depletion of lymphocytes (LD) (**h**). After 15 days of recovery period, mild hypertrophy of tunica media (HT), lymphocytic infiltration (LI), and hyperplasia of the lymphoid follicle (HL)were observed (**i**).

Normal histological structure of spleen sections was noticed in the control group (Fig. 5a). Our histopathological results revealed obvious lesions in the spleen in all treated groups. After CA exposure, there was marked hypertrophy of tunica media accompanied by mild vacuolation, hyperplasia of the lymphoid follicle, and also mild exhaustion of lymphocytes. In this group, melanomacrophage centers (MMC) aggregation was noticed with hemosiderine pigment precipitation in a mild degree (Fig. 5b). On the other hand, severe dilatation and congestion in blood vessels, beginning of thrombus formation, and mild hemorrhage in red pulp also noticed in Clavulanic group (Fig. 5c). After exposure to both sizes of PE-NPs groups almost all histopathological lesions were observed in a moderate to severe degree. Severe intimal and trabecular thickening (connective tissue proliferation) and medial hypertrophy of splenic blood vessels, which resulted in increased portal pressure and development of fibrosis with much vacuolation, were observed clearly in these groups (Fig. 5d). Also, MMC aggregation were detected increasingly in number and size, excessive infiltration of lymphocytic cells accompanied with a multifocal area of depletion of lymphocytes were the most diagnostic features and appeared obviously after exposure to both sizes of PE-NPs groups (Fig. 5f). After exposure to CA and both sizes of PE-NPs, massive infiltration of lymphocytes was detected especially perivascular infiltration. Severe degrees of hemorrhages were diagnosed in red pulp with a multi-focal area of hyperplasia of the lymphoid follicle (Fig. 5e). Both combined groups established with severe exhaustion and depletion of lymphocytes and hypertrophy of tunica media with massive vacuolation (Fig. 5g). Finally, beginning of thrombus formation was noticed in a mild degree in two combination groups of CA and PE-NPs (Fig. 5h). After recovery period in all groups the histopathological remarks were obvious in a moderate to severe degree as the same as exposed groups degree (Fig. 5i).

**Fig. 6:**
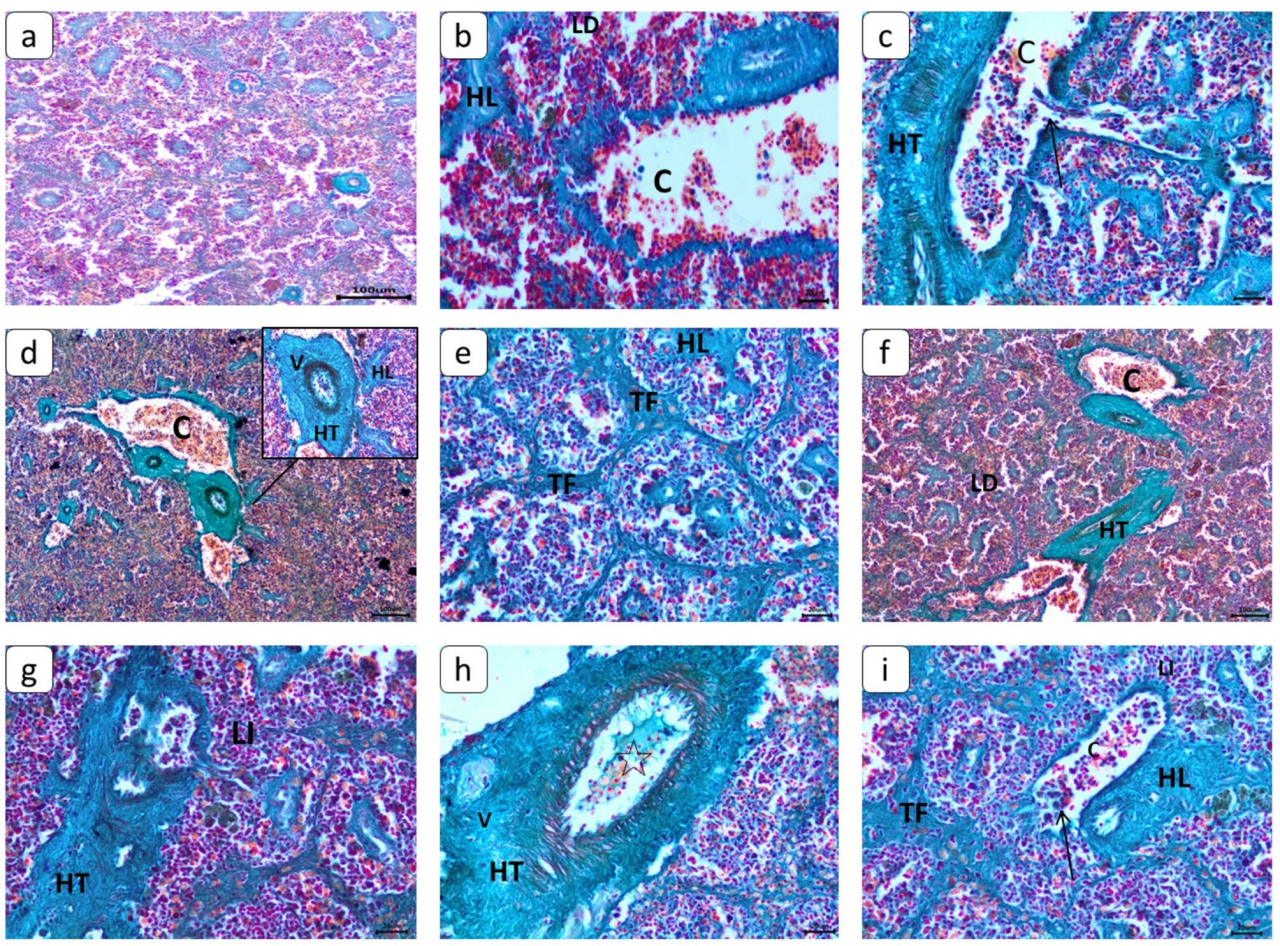
Photomicrograph of *C. gariepinus* spleen (Sirius red staining), showing the normal histological structure of the control group (**a**). Collagenous fibers proliferation in red color around blood vessels (HT), degeneration of blood vessels (arrow) and congestion (C) in CA (100 ng L⁻¹) group (**b** and **c**). Epithelial degeneration of the blood vessel wall (arrow), congestion (C), perivascular collagenous fiber proliferation (HT), hemorrhages in the red pulp (H), and massive trabecular fibrosis (TF) in (20 nm, 5 mg L⁻¹) & (80 nm, 5 mg L⁻¹) of PE-NPs groups (**d** and **e**). Broad distribution of collagenous fibers proliferation around blood vessels (HT) with massive degeneration (arrow), congestion (C), vacuolation (V), and hemorrhages in the red pulp (H) in CA and PE-NPs of both sizes groups (**f**, **g** and **h**). Moderate remarks appeared with few threads of collagenous fibers, congestion (C), hemorrhages (H), and degeneration in its wall (arrow) after recovery period in all groups (**i**).

**Fig. 7:**
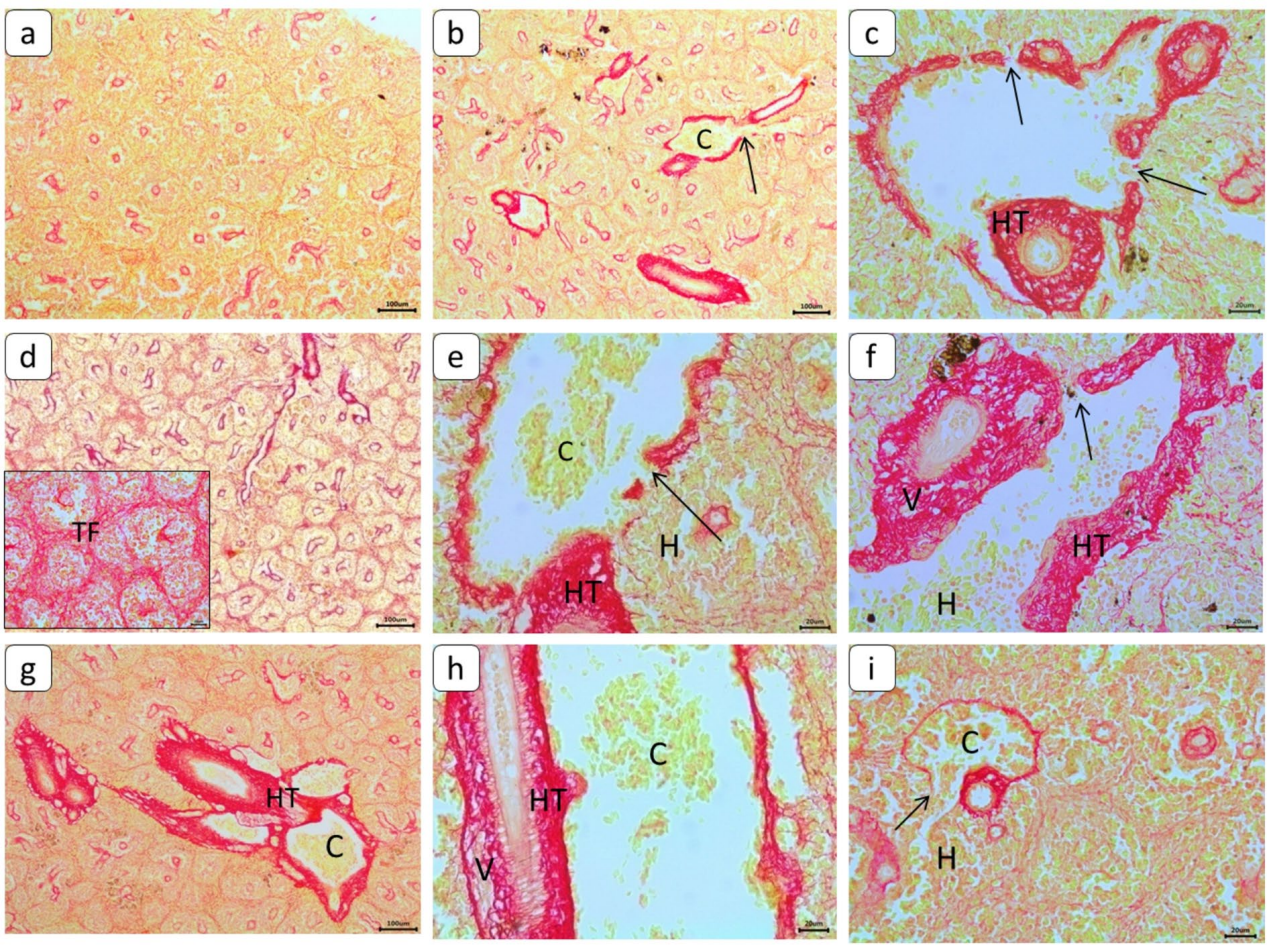
Photomicrograph of *C. gariepinus* spleen (Masson Trichrome’s staining), showing normal histological structure of control group (**a**). hypertrophy of tunica media (HT), hyperplasia of lymphoid follicle (HL), lymphocytic depletion (LD), dilatation and congestion (C) of blood vessels and degeneration of its wall (arrow) in CA (100 ng L⁻¹) group (**b** and **c**). After exposure to (20 nm, 5 mg L⁻¹) & (80 nm, 5 mg L⁻¹) of PE-NPs groups, fibrosis of trabeculae (TF), hyperplasia of the lymphoid follicle (HL), and hypertrophy of tunica media (HT) with vacuolation (V) (**d** and **e**). after exposure to CA and PE-NPs of both sizes groups, depletion of lymphocytes (LD), hypertrophy of tunica media (HT) with massive vacuolation (V), and perivascular infiltration of lymphocytes (LI) (**f** and **g**). Thrombosis (star) and hyperplasia of the lymphoid follicle (HL) and hypertrophy of tunica media (HT) with massive vacuolation (V) (**h**). After the recovery period, hyperplasia of the lymphoid follicle (HL), congestion of blood vessels (C) with degeneration of its wall (arrow), mild trabecular fibrosis (TF), and lymphocytic infiltration (LI) in mild to moderate degree (**i**).

Spleen sections stained with Masson trichrome and Sirius red stains were used as indicators for collagen fibers (Figs. 6 and 7). Fine radiation of collagenous fibers in red color distributed normally around blood vessels in the control group. While other exposed groups showed more diffuse perivascular capsular fibrosis of collagenous fibers with degeneration of blood vessel walls. Connective tissue proliferation of collagenous fibers was cleared in splenic trabeculae. Sirius red’s stain in spleen sections showed a green color as an indicator for collagen fibers. All treated groups showed massive deposition of collagenous fiber, mainly around blood vessels and a faint network around small blood vessels and in splenic trabeculae. Splenic Vein Aneurysm (SVA) causes medial hypertrophy of splenic blood vessels, which resulted in thickening and clogged blood vessels, which results in increased portal pressure and the development of fibrosis and aneurysm formation. On the other hand, after recovery period, collagenous fibers were in a moderate degree in its deposition. The scoring of histological lesions for different groups is cleared in Table 3.

**Table 1:** Hematological parameters of *C. gariepinus* after 15-day exposure to CA (100 Ng L⁻¹), PE-NPs (5 mg L⁻¹; 20–80 nm), and their combinations.

Hemato-biochemical parameters	Exposure period					
	Control	CA	PE-NPs (20 nm)	PE-NPs (80 nm)	CA + PE-NPs (20 nm)	CA + PE-NPs (80 nm)
RBCs (×10⁶ µL⁻¹)	3.2 ± 0.05	3.42 ± 0.2	2.63 ± 0.1**	2.77 ± 0.03*	2.50 ± 0.1***	2.63 ± 0.04**
Hb (g dL⁻¹)	9.40 ± 0.35	8.48 ± 0.14	7.23 ± 0.17***	7.66 ± 0.34***	6.85 ± 0.16***	7.45 ± 0.28***
HCT (PCV) %	35.85 ± 0.19	33.65 ± 0.27***	31.63 ± 0.344***	33.27 ± 0.52***	29.63 ± 0.21***	31.03 ± 0.18***
MCV (µm^3^)	112.1 ± 1.29	100.5 ± 6.79	120.5 ± 3.23	120.4 ± 2.8	119.1 ± 3.7	118.0 ± 2.05
MCH (Pg)	29.34 ± 0.76	25.21 ± 1.37	27.51 ± 0.76	27.69 ± 1.07	27.48 ± 0.82	28.28 ± 0.87
MCHC (g dL⁻¹)	26.20 ± 0.85	25.22 ± 0.49	22.88 ± 0.69	23.08 ± 1.12	23.13 ± 0.63	24.0 ± 0.87
Platelets (×10³ µL⁻¹)	225.5 ± 4.14	216.7 ± 3.62	205.3 ± 1.31***	210.0 ± 2.13**	197.2 ± 1.58***	198.7 ± 1.74***
WBCs (×10³ µL⁻¹)	11.71 ± 0.26	11.33 ± 0.18	10.33 ± 0.15*	10.90 ± 0.14	9.67 ± 0.21***	9.73 ± 0.49***
Neutrophils%	11.33 ± 0.21	13.33 ± 0.33*	15.0 ± 0.3***	14.50 ± 0.50***	15.7 ± 0.31***	13.67 ± 0.56**
Large lymphocytes%	58.0 ± 0.44	56.0 ± 0.44*	54.50 ± 0.43***	54.83 ± 0.48***	54.17 ± 0.48***	52.33 ± 0.49***
Small lymphocytes%	25.50 ± 0.56	23.83 ± 0.48*	21.33 ± 0.21***	21.50 ± 0.22***	21.50 ± 0.22***	21.17 ± 0.31***
Monocytes%	2.83 ± 0.17	4.50 ± 0.56**	2.17 ± 0.17	2.0 ± 0.26	2.17 ± 0.17	2.0 ± 0.26
Eosinophils%	2.33 ± 0.22	4.50 ± 0.56**	7.0 ± 0.36***	7.16 ± 0.30***	7.0 ± 0.36***	7.50 ± 0.34***

Data are presented as mean ± standard error (SE) (*n* = 6). **Statistical significance: *p < 0.05, **p < 0.01, ***p < 0.001 vs. control.

**Table 2:** Hematological parameters of *C*. *gariepinus* after a 15-day recovery period following exposure to CA (100 Ng L⁻¹), PE-NPs (5 mg L⁻¹; 20–80 nm), and their combinations.

Hemato-biochemical parameters	Recovery period					
	Control	CA	PE-NPs (20 nm)	PE-NPs (80 nm)	CA + PE-NPs (20 nm)	CA + PE-NPs (80 nm)
RBCs (×10⁶ µL⁻¹)	3.3 ± 0.1	3.63 ± 0.18	2.7 ± 0.1**	3.08 ± 0.03	2.60 ± 0.1***	2.95 ± 0.03
Hb (g dL⁻¹)	9.57 ± 0.43	9.43 ± 0.18	7.56 ± 0.19**	8.3 ± 0.41	7.18 ± 0.17***	8.22 ± 0.4
HCT (PCV)%	35.70 ± 0.28	36.9 ± 0.37*	32.95 ± 0.21***	35.9 ± 0.23	31.43 ± 0.19***	34.23 ± 0.31**
MCV (µm^3^)	109.4 ± 1.78	103.0 ± 5.83	123.3 ± 3.56	116.5 ± 1.55	121.4 ± 3.86	116.3 ± 1.98
MCH (Pg)	29.08 ± 0.92	26.20 ± 1.09	28.27 ± 0.83	26.90 ± 1.26	27.69 ± 0.73	27.83 ± 1.3
MCHC (g/dL)	26.78 ± 1.05	25.58 ± 0.61	22.98 ± 0.68*	23.11 ± 1.11	22.87 ± 0.66*	23.59 ± 1.01
Platelets (×10³ µL⁻¹)	227.5 ± 6.6	238.2 ± 3.96	216.7 ± 1.78	229.3 ± 1.9	205.3 ± 1.31**	216.5 ± 2.38
WBCs (×10³ µL⁻¹)	11.57 ± 0.27	12.48 ± 0.19*	10.72 ± 0.18	12.08 ± 0.17	10.22 ± 0.18***	11.23 ± 0.19
Neutrophils%	11.50 ± 0.22	13.67 ± 0.21***	14.50 ± 0.22***	13.33 ± 0.42**	14.67 ± 0.33***	14.0 ± 0.36***
Large lymphocytes%	57.50 ± 0.67	58.0 ± 0.58	55.50 ± 0.22	57.33 ± 0.33	55.5 ± 0.67	55.67 ± 0.33
Small lymphocytes%	25.67 ± 0.49	23.0 ± 0.52**	22.17 ± 0.40***	21.50 ± 0.43***	21.67 ± 0.33***	21.83 ± 0.47***
Monocytes%	2.83 ± 0.17	4.50 ± 0.56**	2.17 ± 0.17**	2.0 ± 0.26***	2.17 ± 0.17**	2.0 ± 0.26**
Eosinophils%	2.33 ± 0.21	3.17 ± 0.79	5.8 ± 0.31***	6.0 ± 0.36***	6.17 ± 0.31***	6.33 ± 0.33***

Data are presented as mean ± standard error (SE) (*n* = 6). **Statistical significance: *p < 0.05, **p < 0.01, ***p < 0.001 vs. control.

**Table 3:** Spleen lesion scores of *C. gariepinus* during the exposure and recovery periods following treatment with CA (100 ng L⁻¹), PE-NPs (5 mg L⁻¹; 20–80 nm) both individually and Their combination.

Spleen Lesions	CA		PE-NPs (20 nm)		PE-NPs (80 nm)		CA + PE-NPs (20 nm)		CA + PE-NPs (80 nm)	
	Exposed	Recovery	Exposed	Recovery	Exposed	Recovery	Exposed	Recovery	Exposed	Recovery
Depletion of lymphocytes	++	+++	++	+++	+++	++++	+++	++++	+++	+++
Lymphocytic infiltration	+++	+++	+++	+	++++	+++	++++	++++	++++	+++
Hyperplasia of lymphoid follicles	++	+++	+	+++	+++	++	+++	+++	++	+++
Fibrosis development Thickened trabiculae	++	+	++	+	++	++++	++	+	+	+
Hypertrophy of tunica media	++++	+++	+++	++++	+++	+++	+++	++++	+++	+++
Melanomacrophages Aggregation	+++	+++	++	++++	+++	++++	++	++	+++	++
Hemosedrine pigment precipitation	++	++	+++	+++	++	++	++	+++	++++	++
Vascular lesions Hemorrhage of red pulp	++	+++	+++	++++	+++	++	+++	+++	+++	+++
Congestion of blood vessels	+++	+++	++++	++	+++	+	++	+++	++	+++
Vacuolation	++	+++	+++	++++	+++	+++	++	+++	+++	+++
Thrombosis	+	+	–	–	–	–	+	–	–	+

The severity of the pathology was assessed using the following scale: (-) for absence, (+) for minimal, (++) for moderate, (+++) for severe, and a maximum score of (++++).

## Discussion

Plastic pollution is a growing global concern because NPs interact with biomolecules and contaminants. When combined with pharmaceuticals, NPs may modify transport, bioavailability, and toxicity through the Trojan horse mechanism^13–15^.

The present study provides the first comprehensive evaluation of the combined toxicity of PE-NPs and CA in *C. gariepinus*, a widely used freshwater fish model. The results demonstrate that CA and PE-NPs (20 and 80 nm), both individually and in combination, induce genotoxic, hematotoxic, and histopathological alterations, with co-exposures—particularly involving 20 nm particles—producing the most severe and persistent effect.

### Erythrocyte apoptosis

In this study, Acridine orange staining reveals a significant increase in apoptosis in *C. gariepinus* erythrocytes exposed to PE-NPs (20 nm), whereas exposure to CA or PE-NPs (80 nm) shows non-significant differences compared to the controls. The combination of CA with 20–80 nm PE-NPs significantly enhances erythrocyte apoptosis compared with the control (*p* < 0.001), with the 20 nm particles inducing a more pronounced pro-apoptotic effect than the 80 nm particles. These results confirm a clear size-dependent enhancement of apoptosis, indicating that smaller PE-NPs intensify CA-induced cytotoxicity.

Our data are consistent with the study by^53–55^ which showed that smaller NPs induce more cytotoxicity. Multiple studies have identified pathways that trigger apoptosis in cells, although the particular details remain unclear. Yirong et al.^56^. found that di (2-ethylhexyl) phthalate promotes apoptosis in carp neutrophils by initiating an oxidative burst. Walpitagama et al.^57^. explained this through ROS-prompted apoptosis, supported by Hamed et al.^58^. Additionally, NPs have been shown to upregulate pro-apoptotic proteins linked to cell apoptosis^56^.

### DNA damage

In this study, the alkaline comet assay reveals that exposure to PE-NPs alone produces non-significant increase in DNA damage in *C. gariepinus* erythrocytes, consistent with previous reports of limited genotoxicity from MPs/NPs in aquatic organisms^59,60^. In contrast, CA alone induces significant DNA damage in *C. gariepinus* erythrocytes, in agreement with previous findings in mollusks^43^. However, co-exposure to CA and PE-NPs (20 nm) significantly elevates DNA damage (1.4-fold vs. control, *p* < 0.05), whereas PE-NPs (80 nm) show non-significant effect in the presence of CA. These findings suggest a size-related pattern of genotoxicity, where smaller particles appear to increase CA-associated DNA damage.

The enhanced DNA damage observed with PE-NPs (20 nm) is likely due to their larger surface area, which increases CA adsorption, cellular uptake, and ROS generation^40,61–64^. While PE-NPs alone cause non-significant DNA effects, they markedly amplify CA toxicity, suggesting a sensitizing role through membrane disruption^65–67^ and facilitation of intracellular CA accumulation. These findings imply that PE-NPs may function both as toxicants and as carriers, potentially enhancing pharmaceutical genotoxicity in a size-dependent way.

Our results are supported by prior studies on NP–antibiotic co-toxicity. For example, polystyrene microplastics were shown to enhance sulfamethoxazole toxicity in zebrafish embryos^32^, and NPs increased the effects of enrofloxacin in *Daphnia magna* by altering life history traits and gut microbiota^33^. Similarly, hematological and hepatic alterations were reported in carp exposed to tetracycline and microplastics^34^, consistent with our findings in *C. gariepinus.* A key novelty of our study is the inclusion of a 15-day recovery phase, which reveals that damage persists or worsens after exposure. This highlights the importance of considering delayed and cumulative toxicity, in line with other reports of long-term risks from NP–pharmaceutical mixtures in aquatic organisms^68,69^.

The persistence and in some cases worsening of toxicological endpoints during the recovery period indicate that CA and PE-NPs may cause prolonged physiological stress that appears only partially reversible. A key contributing factor may be the bioaccumulation and tissue retention of NPs, which have been shown to accumulate in fish liver and muscle and to cause sustained genotoxicity even after chronic exposure^70^. In addition, the Trojan horse effect may prolong CA availability by adsorptive binding to PE-NPs, resulting in delayed release and extended cellular exposure. Co-exposure studies further indicate that polystyrene -NPs can disrupt mitochondrial function, leading to ROS overproduction, loss of membrane potential, and impaired repair capacity^71^. Another contributing factor is delayed or secondary inflammatory activation. NPs and microplastics activate immune signaling cascades such as NF-κB and TLR4/MAPK, which may remain active after exposure ends. This can sustain leukocyte depletion, cytokine upregulation, and tissue lesions. For example, polystyrene-MPs induced broad inflammatory gene expression across multiple tissues in *Channa argus*^72^, while carp studies demonstrated NF-κB–mediated cytokine release and ferroptosis^73^ and myocardial inflammation through TLR4/NF-κB signaling^74^. In seabream, subchronic exposure produced persistent hepatic inflammation and cytokine induction^75^. Moreover, recovery experiments in *Eriocheir sinensis* revealed that immune enzyme activities and cytokine expression did not fully return to baseline, confirming incomplete resolution^76^. Similar findings in zebrafish demonstrated that NPs, through different exposure routes, activated TLR4 pathways and induced inflammatory effects^77^, with additional evidence of oxidative stress–mediated inflammation in neurodevelopmental tissues^78^. Taken together, these findings indicate that the incomplete recovery observed in our study likely reflects a combination of bioaccumulation, mitochondrial dysfunction, and prolonged immune activation, which maintain oxidative and inflammatory stress beyond the exposure window. Such persistence highlights the ecological risk of NP–antibiotic mixtures and underscores the importance of considering delayed effects in aquatic toxicology.

The rise in erythrocyte apoptosis and DNA damage indicates compromised physiological integrity in *C. gariepinus*. Apoptosis reduces oxygen transport, while DNA damage reflects genomic instability and oxidative stress^79,80^. Such cellular injuries can weaken immunity, impair respiration, and hinder growth and reproduction, ultimately affecting population fitness, particularly in benthic species exposed to sediment-associated pollutants^81–83^.

### Hematological alterations

Blood parameters are reliable indicators of fish health and toxicant exposure^84^. In this study, *C. gariepinus* exposed to CA and PE-NPs exhibits marked reductions in Hb, RBCs, Hct, and WBCs, with the most pronounced changes in the co-exposure groups. These alterations indicate anemia and immunosuppression, consistent with previous reports of microplastic- or nanoparticle-induced hematological disturbances in freshwater fish. For example, polystyrene microplastics reduced RBC, Hb, and Hct values in Labeo rohita^85^, while ZnO and TiO₂ nanoparticles decreased erythrocyte counts and altered immune cell profiles^86,87^. Importantly, hematological disturbances in our experiment persist after the recovery period, suggesting incomplete restoration of blood function. Such impairments in oxygen transport and immune defense may reduce survival and resilience in fish populations exposed to repeated contaminant stress.

### Histopathological findings

Histopathology can reveal the presence and severity of abnormalities that may not be visible through biochemical examinations alone. Over the years, the spleen has been examined due to its function in the initiation of adaptive immune responses in many vertebrate species, including fish^88^. Moreover, the spleen plays a crucial role in immunological defense mechanisms, elimination of immune complexes, hematopoiesis, and clearing foreign substances from circulation^89,90^. Sayed et al.^91^ have verified that NPs disrupt antioxidant systems, leading to an overproduction of ROS in the cells of the spleen^92^. MPs can be identified by the immune system as foreign entities, prompting an immunological response that could potentially result in persistent inflammation or other immune-related complications^93^.

Our results of exposing catfish for 15 and recovery after 30 days to CA, PE-NPs, and its combination are distinguished by clear unpleasant pathological Lesions in the spleen, including severe dilatation and congestion of blood vessels in blood vessels and hemorrhage in the red pulp, massive perivascular infiltration of lymphocytes, hyperplasia of lymphoid follicle, depletion of lymphocytes, hypertrophy of tunica media with massive vacuolation and small thrombosis. MMC aggregation was detected increasingly in numbers and sizes with hemosidrosis. Moderate to severe degrees of histopathological remarks remain obvious after the recovery period.

David and Kartheek^88^ results are in agreement with our present investigations, as an occurrence of haemosiderin pigment, a large number of MMC centres, necrotic eosinophils, and vacuolation in the spleen of all freshwater fish *Cyprinus carpio* subjected to a sublethal dose of 0.2 mg/L of sodium cyanide for a period of 10 and 20 days. MMC is a significant immune cell type found in the spleen of teleosts, characterized by its physiological, distinctiveness, and predominance importance^94^. The composition of MMC includes four distinct types of brown pigments: hemosiderin, ceroid, lipofuscin, and melanin pigment, all of which are found within vacuoles. Sayed et al.^95^. determined that the rise in the quantity of MMC might function as an indicator of environmental variables, hazardous effects, and stress. The accumulation of melanin in macrophages in fish serves an important role in neutralizing harmful free radicals that are produced during unsaturated lipid peroxidation^89^.

As a result of previous studies, Zhao et al.^96^. subjected grass carps to SMZ and CMN for 42 days. Subsequently, the carps were exposed to A. hydrophila for 2 days after injection. The histological section of the fish spleen in the CMN, SMN, and MIX groups exhibited an increase in cell vacuolation and the activation of MMC. Research has demonstrated that surrounding pollutants can disrupt the balance between exogenous and endogenous ROS, primarily generated by mitochondria. This disruption can result in oxidative harm to organisms and can lead to apoptosis^97^.

In a similar trend, Bardhan et al.^98^. detected the effect of concentrations of 0.02, 0.4 and 8 mg/kg PE-MPs in the feed of Japanese quail (*Coturnix japonica*) for 5 weeks. Hemosiderosis likely results from cytotoxicity. The dose-related increase in hemosiderin, attributed to erythrocyte destruction, led to congestion in the red pulp trabecula, prominent vacuolation in all splenic sections, along with increased sinusoidal space, all are indicative of precedents of splenic necrosis. Haemosiderosis is a pathological condition caused by the accumulation of haemosiderin, which is associated with an elevated rate of erythrocyte destruction in the spleen. Consequently, this may lead to a reduction in the level of hemoglobin, which is typically caused by the loss of RBCs and the abnormal flow of haemoglobin from the spleen in fish^99^.

The results obtained in this study align with the findings of the presence of vacuoles in the spleen tissue of European eels (*Anguilla anguilla*) as a consequence of stress. This vacuolation led to a disruption of the fish’s normal physiological processes. It may involve immune suppression as a result of a decrease in the number of mature lymphocytes.

On the other hand, Bhuyan^100^ collected Data from many countries around the world concluded that, the thickening of blood vessel walls, hemorrhages, and infiltration of MMC characterize lesions in the spleen. Ali et al.^101^ observed a reduction in lymphocytes and an increase in MMC accumulation in the spleen of fish exposed to EO. Furthermore, the exposure to EO resulted in a reduction in the lymphoid follicle of the spleen, varying amounts of MMC infiltrations, and congestion of the blood sinusoids of the spleen. Earlier studies documented increased sinusoidal space in fish due to pharmaceutical toxicity^102,103^.

However, many similar histological alterations between other fish exposed to various pharmaceuticals and drugs support the understanding that extensive splenic necrosis suggests severe splenotoxicity. The observed vacuolation in fish treated with pharmaceuticals indicate necrotic areas. Literature also correlates the occurrence of vacuoles in the red pulp with cytotoxicity, possibly due toarteriolar congestion, loss of blood supply, and arteriolar atrophy^104^. Arteriolar congestion and necrosis were associated with red pulp hemorrhages, that could be formed from necrotic blood and reticulum bodies.

Our investigations are compatible with Bardhan et al.^98^. , who detect thrombopenia, a dose-dependent increase in white pulp compared to a red pulp, reflecting cellular disorientation, increased sinusoid, and resizing of white pulp, upon dietary different concentrations of PE-MPs on *Coturnix japonica*. It might have hindered the prompt formation of an effective hemostatic plug at arteriolar points, leading to unrepaired vascular defects and hemorrhages. Thrombopenia may result from spleen over-activity, causing hematopoietic depression and increased platelet destruction, which lead to large amounts of slow-moving or stagnant blood.

Conversely, it was observed that the persistence of mild to moderate splenic necrosis and increased sinusoidal space resulted in loosely packed splenic tissues, an increase in lymphocyte hyperplasia of white pulp, increased sinusoidal space, and white pulp proliferation, contributing to the enlargement of the spleenpulp atrophyis also associated with conditions like cadmium treatment and lymphopenic mice exposed to vancomycin and neomycin^105^. In addition, Gu et al.^106^. found an increase in lymphocytes along with white pulp proliferation in the spleen, which resembles our findings. Furthermore, NPs compose the portion of plastic waste that may cause the most severe eco-toxicological issues due to its ability to be absorbed by the biota. It also negatively impacts survival, growth, and metabolism and causes oxidative stress and neurotoxicity^107^.

On the contrary, spleen sections stained with Masson’s trichrome and Sirius red used as indicators for collagen fibers. We reported that the proliferation of collagenous fibers in the splenic trabeculae was diminished, while there was significant deposition of collagenous fibers, predominantly surrounding blood vessels, resulting in vascular narrowing.

In splenic tissue, fibrosis entails the accumulation of fibrin first and collagen subsequently, frequently induced by persistent inflammation, infection, or circulatory abnormalities. Fibrin deposition transpires first upon injury or inflammation, creating a temporary matrix that draws immune cells and fibroblasts. Activated fibroblasts, stimulated by cytokines such as TGF-β, deposit collagen, substituting fibrin and resulting in persistent extracellular matrix accumulation and fibrosis. Oxidative stress, such as that caused by ROS, stimulates fibroblast activation and elevates the production of pro-fibrotic genes. It additionally upregulates TGF-β and NF-κB, hence augmenting collagen synthesis. Markers such as MDA (malondialdehyde), SOD, and GSH are utilized to evaluate oxidative stress levels in fibrotic splenic tissue. Consequently, oxidative stress enhances collagen and fibrin accumulation, expediting fibrosis in the spleen^108^.

Subsequently, the impairment of the immune system is attributed to aquatic organisms^,^ exposure to environmental toxins, leading to a notable decrease in the fish’s ability to defend against pathogen intrusion. Further research is needed to investigate the distinctive splenic response in fish, which plays a crucial role in their immune system. The results acquired in this study may contribute to future research in the field of fish immunotoxicology.

After exposure to toxic substances such as CA or PE-NPs, the administration of the toxic dose may cease; however, the induced damage does not immediately resolve, as tissues require an extended period to regenerate cellular structures and reestablish normal immune homeostasis. In the case of the spleen, which serves both immunological and hematopoietic functions, several mechanisms may account for the persistence of histopathological alterations during the recovery period. One possible explanation is the presence of chronic inflammation, since nanoparticles are poorly degradable and may remain entrapped within splenic tissues for a prolonged time, leading to sustained immune activation and continuous inflammatory stimulation. Another contributing factor is tissue fibrosis, as collagen fiber deposition resulting from inflammation is not easily reversible and necessitates tissue remodeling processes that may extend over several weeks or even months. In addition, vascular injury appears to play a crucial role; pathological changes such as medial hypertrophy and partial thrombus formation can impair normal blood flow, thereby delaying tissue repair and regeneration. Moreover, dysfunction of phagocytic and immune cells is indicated by the prominent accumulation of melanomacrophage centers, suggesting that these cells are still processing residual toxic materials or degraded erythrocytes, reflecting a prolonged detoxification and inflammatory state. Wang et al.^109^. and Hamed et al.^110^. findings are in agreement with These interpretations, who demonstrated that NPs exposure induces persistent oxidative and inflammatory damage that continues even after the cessation of exposure, mainly due to vascular impairment and incomplete tissue regeneration. This could explain why splenic lesions remained apparent after the recovery period in the present study.

The results of this study have considerable implications that extend beyond individual-level toxicity. The enduring presence of DNA damage, apoptosis, hematological changes, and splenic pathology in *C. gariepinus* suggests that aquatic creatures subjected to concurrent NPs and antibiotic contamination may suffer from persistent health issues. These deficits may diminish the resilience of fish populations to environmental stressors, disrupt reproductive success, and modify predator-prey dynamics, ultimately resulting in alterations to aquatic community structures. Furthermore, as *C. gariepinus* is a benthic feeder, these effects may bioaccumulate and bio magnify within food webs, presenting hazards to higher trophic levels, including economically significant species and human consumers. The identified toxicological consequences highlight the pressing necessity for more stringent regulation measures concerning the discharge of plastic and pharmaceutical waste into aquatic ecosystems to safeguard biodiversity and sustain ecosystem services.

## Conclusions

This study reveals that PE-NPs synergistically intensify the toxicity of Potassium Clavulanate in *C. gariepinus*. Using multiple biological endpoints, it demonstrates size-dependent toxicity, likely linked to enhanced bioavailability and oxidative stress. Although conducted under controlled conditions with limited mechanistic data, the findings highlight the need for broader, long-term studies on NP–pharmaceutical interactions. Overall, the work emphasizes incorporating such combined effects into environmental risk assessments to better protect aquatic ecosystems.

## Data Availability

All relevant raw data will be freely available from the corresponding author.
